# The impact of the TyG index and psychosocial factors on depression in elderly non-diabetic patients with atrial fibrillation

**DOI:** 10.3389/fpsyt.2026.1746187

**Published:** 2026-03-03

**Authors:** Jianning Ma, Fang Zhu, Dongmei Ren, Kena Bao, Weilan Yan, Min Liu, Xiangdong Xu

**Affiliations:** 1 Department of Cardiology, Jiading District Central Hospital Affiliated Shanghai University of Medicine & Health Sciences, Shanghai, China; 2 Department of Nursing, Jiading District Central Hospital Affiliated Shanghai University of Medicine & Health Sciences, Shanghai, China; 3 Department of Hospital Infection Control, The Fifth Affiliated Hospital, Sun Yat-sen University, Zhuhai, China

**Keywords:** atrial fibrillation, depression, elderly non-diabetic patients, psychosocial factors, TyG index

## Abstract

**Background:**

Atrial fibrillation (AF), a common arrhythmia in the elderly, often causes complications that severely impact quality of life and survival. Depression is common in AF patients and correlates with AF severity. The triglyceride-glucose index (TyG), a novel metabolic biomarker for cardiovascular disease, has also been linked to depression.

**Methods:**

This retrospective study enrolled 337 elderly non-diabetic AF patients admitted to the Department of Cardiology at Jiading District Central Hospital from August 2024 to August 2025. Patients were divided into depression and non-depression groups according to a Patient Health Questionnaire-9 (PHQ-9) score≥ 5. Baseline characteristics, clinical biomarkers and emotional assessments were compared between groups. Variables with p<0.1 were entered into logistic regression to identify independent predictors of depression.

**Results:**

No significant differences were observed between the depression (n=86) and non-depression (n=251) groups in demographic or clinical characteristics (age, sex, BMI, smoking, alcohol use, or hypertension; all p> 0.05). However, significant group differences were identified in metabolic markers (total cholesterol, LDL, and urea; p= 0.034, 0.033, and 0.009, respectively) and psychological assessments (Pittsburgh Sleep Quality Index [PSQI], Chinese version of the Mini-Mental State Examination [CMMSE], and Social Support Rating Scale [SSRS]; all p< 0.001). Logistic regression analysis identified four potential predictors of depression: lower CMMSE score (OR = 0.859, 95% CI: 0.779–0.949; p= 0.002), lower SSRS score (OR = 0.808, 95% CI: 0.747–0.874; p< 0.001), poor sleep quality (higher PSQI; OR = 1.392, 95% CI: 1.266–1.531; p< 0.001), and higher TyG index (OR = 2.15, 95% CI: 1.042–4.450; p= 0.038). Exploratory stratified analyses revealed that cognitive function (CMMSE) and sleep quality (PSQI) were not significantly associated with the TyG index (both p>0.05), suggesting their independent contributions to depression. For social support (SSRS), TyG index did not differ between depression and non-depression groups in the high-support subgroup (SSRS> 30), but a significant difference was observed in the low-support subgroup (SSRS 20-30; p = 0.002).

**Conclusion:**

This study identifies cognitive function, social support, sleep quality and the TyG index as potential influencing factors for depression in elderly non-diabetic AF patients. Targeted management of these factors may improve mental health and overall prognosis in this population.

## Introduction

Atrial fibrillation (AF) is the most common sustained arrhythmia, characterized by the loss of effective atrial contraction and replacement by chaotic fibrillatory waves, leading to hemodynamic abnormalities and an increased risk of thromboembolism (1, 2). Uncontrolled AF progresses to severe complications including cardiac insufficiency and thromboembolism has become one of the major contributors to the global burden of morbidity and mortality (3, 4), significantly compromising patients’ quality of life. Notably, patients with AF also have an increased risk of psychiatric disorders compared with non-AF individuals (5, 6). Research indicates that depressive symptoms are particularly prevalent as a comorbidity among AF patients, with an incidence rate ranging from 20% to 40% (6–9). These emotional disturbances arise from the constellation of clinical manifestations frequently observed in AF patients, including chest tightness, palpitations, fatigue, tension, and sleep disturbances, which collectively contribute to the development of affective disorders (10, 11). A bidirectional relationship exists between AF and depression (12). Depressive symptom prevalence is elevated in patients with AF, a phenomenon primarily driven by the increased disease burden, systemic inflammatory responses, and autonomic nervous system dysfunction inherent to AF (13–15). Conversely, depressive states may exacerbate the risk of AF onset and progression through psychobiological mechanisms including neuroendocrine dysregulation and enhanced platelet activation (16–18). Collectively, these interconnected pathological processes form a bidirectional pathogenic feedback loop between AF and depression, with metabolic disturbances and psychosocial pathways acting as key mediators underlying this bidirectional relationship (19, 20). The Triglyceride-Glucose (TyG) index has emerged as a clinically significant metabolic marker, calculated from routine fasting triglyceride (TG) and fasting plasma glucose (FPG) measurements (21, 22). This simple yet robust index provides a comprehensive assessment of glycolipid metabolism and demonstrates strong associations with cardiovascular pathophysiology (23, 24). Studies have found that higher TyG indices correlate with increased susceptibility to depressive disorders, anxiety disorders, and stress-related pathologies (25–27). Although current evidence on the association between the TyG index and mood disorders remains limited, the well-established link between metabolic disorders and mental health suggests a plausible correlation between the TyG index and mood disorders. Based on the available evidence, metabolic abnormalities and psychosocial stressors, such as cognitive decline, sleep disorders and insufficient social support, are all independent risk factors for depression (25, 28–30). However, their independent effects have not been confirmed in the specific population of elderly patients with atrial fibrillation. Accordingly, the present study puts forward the hypothesis that the metabolic indicator TyG index and psychosocial factors including cognition, sleep and social support independently influence the risk of depression in elderly patients with atrial fibrillation, which will facilitate the accurate identification of influencing factors for depression in this population.

## Materials and methods

Patients aged 60 years and older diagnosed with AF and hospitalized in the Department of Cardiology at Jiading District Central Hospital from August 2024 to August 2025 were included. A total of 337 non-diabetic AF patients were enrolled. Patients with a PHQ-9 score≥ 5 were assigned to the depression group and the rest were assigned to the non-depression group. A retrospective analysis was conducted using a combination of questionnaire surveys and medical history reviews. Differences in basic information, test indicators, lifestyle behaviors, and emotional assessments between the two groups were compared. Potential risk factors with p< 0.1 were included in logistic regression analysis to identify risk factors affecting depression in elderly non-diabetic AF patients.

### Diagnostic criteria

Atrial Fibrillation: AF was diagnosed according to the 2020 European Society of Cardiology (ESC) Guidelines for the Diagnosis and Management of Atrial Fibrillation (31). The diagnosis was confirmed by at least one 12-lead ECG or 24-hour ambulatory ECG (Holter) demonstrating absence of P waves, irregular fibrillatory waves, and absolutely irregular R-R intervals.

Diabetes (Type II):The diagnosis of diabetes is based on the WHO Diagnostic Criteria for Diabetes Mellitus (2023 Update), which specify the following diagnostic thresholds: fasting plasma glucose (FPG) at ≥7.0 mmol/L, 2-hour plasma glucose after an oral glucose tolerance test (OGTT 2hPG) at ≥11.1 mmol/L, random plasma glucose at ≥11.1 mmol/L (required to be accompanied by typical diabetic symptoms) and glycated hemoglobin (HbA1c) at ≥6.5%. Individuals with typical symptoms (polyuria, polydipsia, polyphagia, and unexplained weight loss) can be diagnosed if they meet any of these indicators, while those without symptoms require repeat testing for confirmation.

### Inclusion criteria​

a) Patients who presented specifically for treatment of AF were diagnosed according to the diagnostic criteria outlined in the 2020 ESC Guidelines for the Diagnosis and Management of Atrial Fibrillation; b)Age≥60 years; c) Clinically stable with sufficient baseline data available for evaluation; d) The individuals should be fully conscious, who can communicate clearly and complete questionnaires on their own. e) All participants provided written informed consent.

### Exclusion criteria​

a) Diabetes mellitus; b) Moderate to severe anxiety (SAS score ≥60); c) Active pulmonary disease, severe hepatic/renal dysfunction, or ​marked coagulopathy; d) Other arrhythmias: sick sinus syndrome, ​complete (third-degree) AV block; e) History of psychiatric disorders; f) Current oral corticosteroids or antipsychotics; g) Language or comprehension barriers to completing questionnaires; h) Insufficient baseline data in prior records.

### Emotional disorder assessments

Depression Status: The PHQ-9 depression screening tool was used to assess the depression status, with scores of 0-4 indicating no depression, 5-9 mild depression, 10-14 moderate depression, 15-19 moderate-to-severe depression, and 20-27 severe depression (32, 33).

Anxiety Status: The Zung Self-Rating Anxiety Scale (SAS) was used to evaluate the anxiety status, with severity graded into four levels (scores 1-4). Patients answered 20 questions based on their actual conditions, and the total raw score was multiplied by 1.25, with the integer part taken as the standard score. A SAS standard score≥ 50 indicates the presence of anxiety (34).

Cognitive Dysfunction: Cognitive function was evaluated using the Chinese version of the Mini-Mental State Examination (CMMSE), culturally adapted by Professor Zhang Mingyuan (35–37). Scores of 27-30 denote normal cognitive performance, whereas scores< 27 indicate cognitive dysfunction.

Sleep Quality: The Pittsburgh Sleep Quality Index (PSQI) evaluates overall sleep status over a period. Higher scores indicate poorer sleep quality, with a cutoff value of 5, demonstrating high sensitivity and specificity (38).

### Clinical test indicators

The following biochemical and hematological parameters were measured: alanine aminotransferase (ALT), aspartate aminotransferase (AST), creatinine, urea, glycated hemoglobin (HbA1c), hemoglobin (Hb), potassium(K), sodium (Na), chloride (Cl), platelets (PLT), international normalized ratio activated (INR), partial thromboplastin time (APTT), prothrombin time (PT), D-Dimer, thyroid-stimulating hormone (TSH), free triiodothyronine (FT3), free thyroxine (FT4), triglycerides (TG), total cholesterol (TC), high-density lipoprotein (HDL), low-density lipoprotein (LDL),fasting blood-glucose (FBG), creatinine(Cr) and blood urea nitrogen(BUN).

A fasting venous blood sample of 4 mL was collected in the morning using a biochemical vacuum tube. The serum was separated within 2 hours at room temperature. All tests were performed using an Abbott ARCHITECT c16000 automated biochemical analyzer, with all procedures strictly followed according to the standard operating protocols.

### Triglyceride-glucose index

TyG Index = Ln[TG×FPG/2], where TG is in (mg/dL) and FBG is in (mg/dL). Unit conversion: 1 mmol/L FBG = 18.02 mg/dL; 1 mmol/L TG = 88.545 mg/dL.

### Statistical methods

All collected data were analyzed using STATA 12.0 statistical software. Normally distributed measurement data were expressed as mean ± standard deviation (Mean ± SD) and compared using t-tests. Skewed data were described using the Median (IQR) and analyzed using the Mann-Whitney U test. Categorical data were presented as percentages (%) and statistically analyzed via chi-square tests or Fisher’s exact test. A p-value < 0.05 was considered statistically significant. Logistic regression analysis was used to identify risk factors for emotional disorders in elderly non-diabetic AF patients.

## Results

### Comparison of basic information and clinical indicators

Comparative analysis of patient demographics and test results revealed statistically significant differences between the depression group (n=86) and the non-depression group (n=251) in several clinical and demographic variables (**Table 1**). No statistically significant differences were found in basic information, including age, gender, BMI, smoking, alcohol consumption, or hypertension status (p>0.05). However, significant differences were observed in test indicators, particularly total cholesterol, LDL, and urea levels (p=0.034, p=0.033 and p=0.009, respectively). In emotional disorder-related assessments, significant differences were found in PSQI, CMMSE, and SSRS scores between the two groups (p = 0.000, p = 0.000, and p = 0.000, respectively).

**Table 1:** General information and clinical characteristics of the study population.

Parameter	Depression group (n=86)	Non-depression group (n=251)	t/χ^2^	p
Age	77.802 ± 7.203	76.124 ± 7.659	-1.781	0.076
Gender(male/female)	41/45(47.67%/52.33%)	129/122(51.39%/48.61%)	0.355	0.552
BMI	23.177 ± 4.023	23.863 ± 3.768	1.432	0.153
Smoke	13(15.12%8)	40(15.94%)	0.0325	0.857
Drink	5((5.81%)	13(5.18%)	0.051	0.821
HBP	51(59.30%)	143(56.97%)	0.142	0.706
Hb	129.882 ± 18.374	128.516 ± 16.912	-0.629	0.530
InR	1.060 ± 0.222	1.085 ± 0.292	0.705	0.482
APTT	28.611 ± 4.609	28.888 ± 4.779	0.467	0.641
PT	12.471 ± 2.480	12.557 ± 2.542	0.273	0.785
D2	1.611 ± 6.418	0.776 ± 1.510	-1.892	0.059
TC*	4.178 ± 1.078	3.882 ± 1.121	-2.129	0.034
HDL	1.211 ± 0.352	1.187 ± 0.336	-0.554	0.580
LDL*	2.433 ± 0.912	2.186 ± 0.926	-2.137	0.033
TSH	3.112 ± 6.549	3.061 ± 6.542	-0.061	0.952
FT3	3.682 ± 0.982	3.938 ± 1.393	1.531	0.127
FT4	14.004 ± 3.499	14.139 ± 2.895	0.343	0.732
PLT	171.372 ± 54.835	170.574 ± 59.832	-0.109	0.913
K	4.001 ± 0.419	3.993 ± 0.494	-0.133	0.894
Na	140.407 ± 3.244	140.344 ± 3.507	-0.146	0.884
Cl	106.942 ± 4.484	106.664 ± 3.844	-0.553	0.580
HbA1c	6.309 ± 0.994	6.235 ± 0.980	-0.590	0.556
ALT^#^	16.20 (11.20, 24.70)	16.30 (12.00, 23.60)	0.003	0.997
AST^#^	19.60 (16.10, 28.80)	21.00 (17.40, 27.00)	0.121	0.262
Cr	90.335 ± 29.792	83.718 ± 30.549	-1.744	0.082
BUN*	7.413 ± 4.113	6.424 ± 2.519	-2.622	0.009
TG	1.352 ± 0.822	1.239 ± 0.777	-1.145	0.253
FBG	5.923 ± 4.009	5.474 ± 1.566	-1.480	0.140
PSQI*	7.244 ± 5.108	2.884 ± 3.106	-9.386	0.000
CMMSE*	22.070 ± 5.539	25.008 ± 3.255	5.937	0.000
SSRS*	28.419 ± 4.912	32.765 ± 4.402	7.667	0.000
TyG	8.563 ± 0.623	8.438 ± 0.576	-1.696	0.091

*The difference was statistically significant.

#Data with skewed distribution were analyzed using the Mann-Whitney U test.

### Identification of factors related to depression in elderly non-diabetic AF patients

To identify factors related to depression, logistic regression analysis was performed. Depression status was used as the outcome variable, and parameters with p< 0.1 from the comparative analysis were included as independent variables. The final analysis included age, D-dimer, total cholesterol, LDL, creatinine, urea, PSQI, CMMSE, SSRS, and TyG index. As shown in **Table 2**, CMMSE score was negatively correlated with depression risk (OR = 0.859, 95% CI: 0.779–0.949, p = 0.002), indicating that better cognitive function was associated with lower depression risk. SSRS score was also negatively correlated with depression risk (OR = 0.808, 95% CI: 0.747–0.874, p = 0.000), suggesting that better social support reduced depression risk. PSQI was positively correlated with depression risk (OR = 1.392, 95% CI: 1.266–1.531, p= 0.000), suggesting a significant potential association between sleep quality and depressive symptoms. The TyG index was positively correlated with depression risk (OR = 2.15, 95% CI: 1.042–4.450, p = 0.038), suggesting that higher TyG levels increased depression risk. Age, D-dimer, total cholesterol, urea, and creatinine levels showed no significant correlation with depression risk (p = 0.619, p = 0.795, p = 0.089, p = 0.453, and p = 0.893, respectively).

**Table 2:** Identification of relevant factor for depression in elderly Non-Diabetic AF Patients.

Parameter	OR	95% CI	z	p
Age	1.014	0.961, 1.069	0.50	0.619
D-Dimer	0.978	0.828, 1.155	-0.26	0.795
BUN	1.059	0.913, 1.228	0.75	0.453
Cr	0.999	0.987, 1.011	-0.13	0.893
TC	0.478	0.204, 1.119	-1.70	0.089
LDL	2.490	0.944, 6.568	1.84	0.065
PSQI*	1.392	1.266, 1.531	6.83	0.000
CMMSE*	0.859	0.779, 0.947	-3.06	0.002
SSRS*	0.808	0.747, 0.874	-5.35	0.000
TyG*	2.153	1.042, 4.450	2.07	0.038

*The difference was statistically significant.

### Exploratory stratified analysis

**Figure 1:**
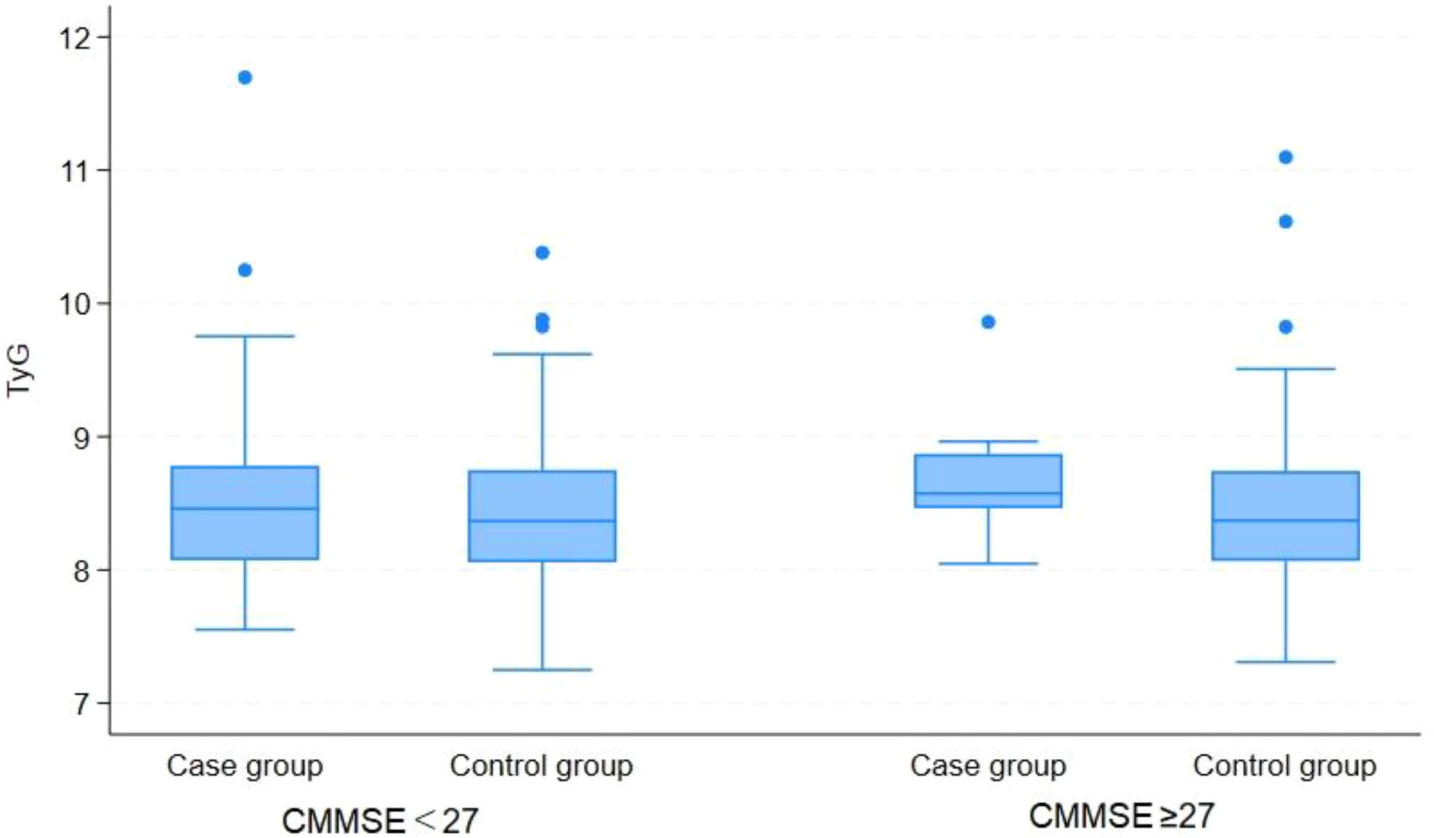
Comparison of TyG index between depressed and non-depressed participants, stratified by cognitive function level.

**Figure 2:**
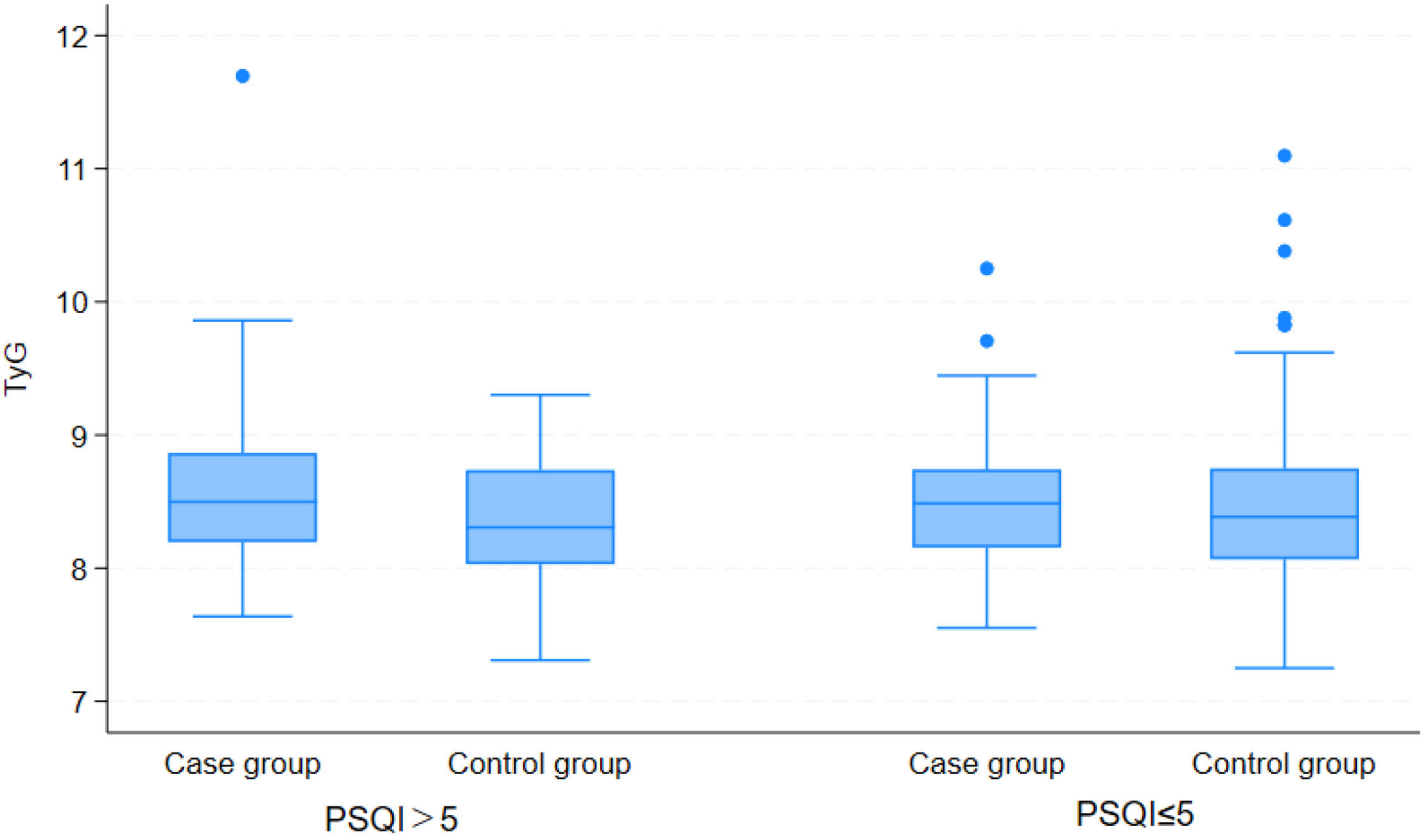
Comparison of TyG Index between depressed and non-depressed participants, stratified by sleep quality level.

**Figure 3:**
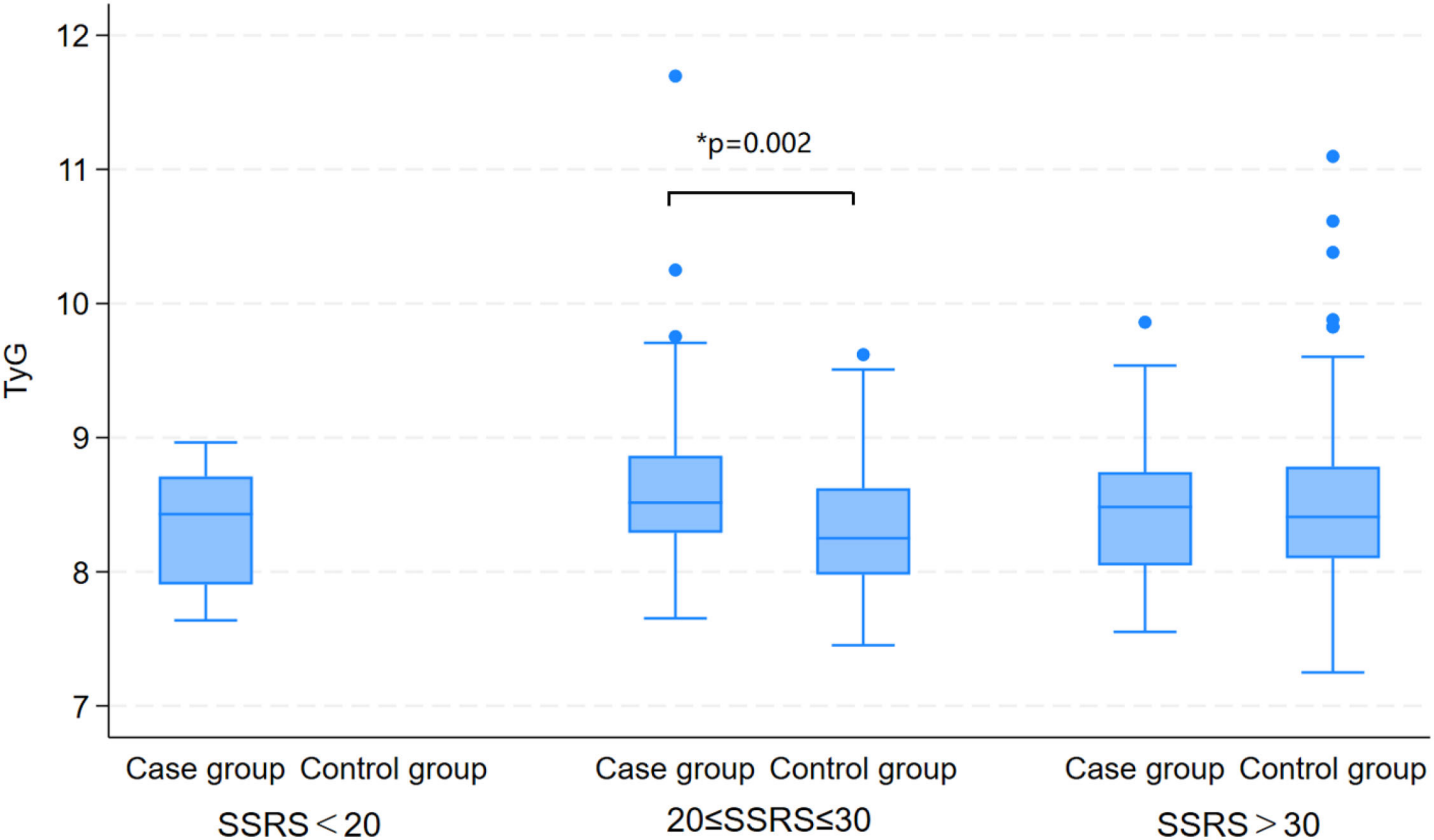
Comparison of TyG index between depressed and non-depressed participants, stratified by social support level. The number of cases in the control group with SSRS < 20 was 0, thus no statistical analysis was performed for this subgroup.

Exploratory stratified analyses were conducted to evaluate potential variations in the TyG index across strata categorized by cognitive function (CMMSE), sleep quality (PSQI), and social support (SSRS), thereby elucidating the associations between these psychosocial factors and metabolic status. The corresponding results are presented in **Table 3**; **Figures 1**–**3**. Stratified analyses by CMMSE revealed no significant between-group difference in the TyG index (p>0.05), suggesting no meaningful association between cognitive function and glucose-lipid metabolism. Furthermore, cognitive function appears to affect depression independently of the TyG index. PSQI stratification showed no significant difference in the TyG index between the good and poor sleep quality groups (p> 0.05), indicating no association between sleep quality and glucose-lipid metabolism. Sleep problems and metabolic dysregulation may independently contribute to the risk of depression in elderly, non-diabetic AF patients. SSRS stratification revealed no significant between-group difference in the TyG index between depressed and non-depressed participants with adequate support (SSRS> 30, p> 0.05), whereas the difference reached statistical significance in the relatively insufficient support subgroup (SSRS 20-30, p= 0.002).

**Table 3:** Stratified analysis results of CMMSE, PSQI and SSRS.

Parameter	Depression group	Non-depression group	t	p
CMMSE				
<27	8.540 ± 0.662	8.427 ± 0.560	-1.344	0.180
≥27	8.663 ± 0.409	8.461 ± 0.611	-1.264	0.210
PSQI				
>5	8.594 ± 0.672	8.353 ± 0.491	-1.945	0.055
≤5	8.520 ± 0.553	8.456 ± 0.592	-0.602	0.548
SSRS				
<20	8.329 ± 0.551	–	–	–
20~30	9.652 ± 0.692	9.308 ± 0.477	-3.127	0.002
>30	8.475 ± 0.089	8.485 ± 0.602	0.089	0.929

## Discussion

In elderly non-diabetic patients with atrial fibrillation, factors significantly affecting depression are not limited to traditional demographic characteristics and clinical factors, while the associations of psychosocial and metabolic factors with depression are gradually being confirmed (39–41). This study investigates the separate and joint effects of the TyG index with psychosocial factors on depression risk in this population, which is aimed to explore whether these factors predict depression better than conventional factors. Through retrospective analysis of 337 elderly non-diabetic AF patients, several key findings were obtained, which provide critical insights into the understanding and clinical management of emotional disorders in elderly AF patients.

Univariate analysis revealed no statistically significant differences in general demographic and basic clinical characteristics between the depressed and non-depressed groups, including age, gender, BMI, smoking, alcohol use history and hypertension prevalence (all p > 0.05). This suggests that among elderly non-diabetic atrial fibrillation patients, depression was not significantly influenced by traditional baseline factors, which is consistent with the conclusions of a middle-aged and elderly CVD study by Xu et al. (42). Typically, factors such as age, gender, and BMI are considered as potential influencing factors for depression in the general population, but this study did not find such an association. It is speculated that the core reason stems from the unique characteristics of the study cohort, where all elderly non-diabetic atrial fibrillation patients have underlying cardiovascular lesions and are generally older. For such populations, the occurrence of depression may be more regulated by specific factors such as disease-related metabolic disorders, changes in cognitive function, and psychosocial support, rather than conventional demographic or basic disease characteristics. Meanwhile, this result eliminates the confounding effect of baseline factors on the key results of this study, which thereby enhances the reliability and accuracy of subsequent analyses by exploring associations between laboratory indicators, scale scores, and depression. For laboratory indicators, most results showed no statistically significant differences between the two groups, including hemoglobin, coagulation function, thyroid function, complete blood count, electrolytes, glycated hemoglobin, liver function, creatinine, triglycerides, and fasting blood glucose. These findings suggest that these indicators may not be significantly associated with depression onset in elderly non-diabetic atrial fibrillation patients, which highlights that influencing factors in this population are primarily concentrated in metabolic and renal function indicators. Notably, the two groups differed significantly in three indicators, namely TC, LDL and BUN (all p<0.05), and the depressed group exhibits the highest levels across all three groups. Specifically, the depressed group showed marked elevation in TC and LDL (key markers of lipid metabolism abnormalities), suggesting that lipid metabolism disorders may be associated with depression onset in elderly non-diabetic atrial fibrillation patients. Previous researches have demonstrated that lipid metabolism abnormalities contribute to negative emotions and depression risk by disrupting brain neurotransmitter homeostasis, driving systemic inflammation and intensifying oxidative stress (43, 44). However, it is important to note that while TC and LDL levels differed significantly between groups in this study. Whether they act as independent risk factors for depression in this population or not requires further validation via multivariate regression analysis. This is because lipid metabolism indicators are interconnected, and differences in a single indicator may be indirectly confounded by other metabolic factors. In addition, TG levels did not differ significantly between the two groups (P = 0.253), which is contrary to some previous studies (45, 46). This may reflect the strict restriction of the study cohort to non-diabetic individuals, which likely minimizes confounding from diabetes-related glucose metabolism disorders in the TG-depression link. The depressed group had significantly higher BUN levels than the non-depressed group (P = 0.009), suggesting that mild renal metabolic disturbances may be closely associated with depression onset in this population. As a common marker of renal function, elevated BUN typically reflects glomerular filtration impairment or abnormal protein metabolism. Notably, elderly atrial fibrillation patients often have cardiovascular comorbidities and are susceptible to reduced renal perfusion, which can elevate BUN levels (47). The speculated mechanism is that mild renal dysfunction reflected by high BUN exacerbates systemic inflammation and oxidative stress, which could impair the secretion of antidepressant neurotransmitters in the brain. Additionally, physical discomfort from abnormal renal function enhances the psychological burden in elderly patients, triggering depressive symptoms (48). This aligns with previous findings that renal dysfunction is linked to a higher risk of depression in older adults (49, 50). Importantly, univariate analysis showed no statistically significant difference in the TyG index between the two groups (P = 0.091). However, the p-value approached the significance threshold of 0.05, indicating a trend toward a higher TyG index in the depressed group compared to the non-depressed group. Prior meta-analyses and cohort studies confirmed that an elevated TyG index is significantly positively correlated with depression risk and served as a potential metabolic marker for depression onset (51, 52). Regarding mood disorder-related scale scores, the two groups exhibited highly significant differences in PSQI, CMMSE, and SSRS scores (all p = 0.000). These findings further corroborate the core roles of sleep quality, cognitive function and social support in depression development among elderly non-diabetic atrial fibrillation patients (53–55). Specifically, the depressed group exhibited significantly higher PSQI scores than the non-depressed group. This indicates poorer sleep quality in the depressed group, which is consistent with prior research findings. However, the relationship between sleep disorders and depression is bidirectional. On one hand, depressive emotions can lead to difficulties in sleep initiation and maintenance. On the other hand, long-term poor sleep quality exacerbates negative emotions and accelerates depression onset and progression. In elderly atrial fibrillation patients, it may also worsen physical discomfort, further deteriorating depressive states (29, 56). The depressed group exhibited significantly lower CMMSE scores than the non-depressed group. This indicates that impaired cognitive function is closely linked to depression onset in this population. Elderly atrial fibrillation patients are prone to cognitive decline due to insufficient cerebral blood supply and age-related degenerative changes. Impaired cognitive function can increase the risk of depression by reducing negative cognitive bias, lowering patients’ ability to recognize diseases and cope with stress (28). Meanwhile, depressive emotions can further impair cognitive function by creating a bidirectional negative cycle. This correlation has been confirmed by multiple studies in elderly cardiovascular disease patients. Furthermore, the depressed group exhibited significantly lower SSRS scores than the non-depressed group, indicating that insufficient social support is a key risk factor for depression in this population. Adequate social support has an antidepressant protective effect by buffering psychological stress, mitigating disease related loneliness and regulating hypothalamic pituitary adrenal (HPA) axis function (57, 58). Limited mobility and reduced social interactions are common among elderly atrial fibrillation patients. These conditions often lead to diminished social support, thereby increasing their vulnerability to depressive symptoms (59).

Based on the univariate analysis, we conducted multivariate Logistic regression analyses by adjusting for age, coagulation function, hepatic and renal function, and lipid profiles to explore independent factors associated with depression in elderly non-diabetic patients with AF. The PSQI appeared to be the most prominent independent risk factor for depression (OR = 1.392, 95%CI: 1.266~1.531, P = 0.000), which suggests that poor sleep quality may independently and notably raise the risk of depression in this group. This finding indicate that sleep disturbance is not merely a concomitant symptom of depression but may act as a core predictor in these patients. By disrupting central neurotransmitter balance and activating the HPA axis, it is likely to contribute to depressive pathology, potentially forming a vicious cycle with AF-related sleep disruption (57, 60). Improving sleep quality may be an effective strategy for preventing and managing depression in AF patients (61). Our study also found that the CMMSE (OR = 0.859, 95%CI: 0.779~0.947, p=0.002) and SSRS (OR = 0.808, 95%CI: 0.747~0.874, P = 0.000) were potential protective factors for depression. Optimal cognitive function enhances emotional regulation and stress coping, while adequate social support mitigates disease-related anxiety. Together, these factors reduce the risk of depression and identify key targets for clinical intervention (30, 62). Furthermore, the triglyceride-glucose (TyG) index, a surrogate marker for insulin resistance, has been proved to be an independent risk factor for depression (OR = 2.153, 95%CI: 1.042~4.450, P = 0.038). This finding highlights a significant association between insulin resistance and depression among elderly non-diabetic patients with AF. This association may operate through chronic inflammation and aberrant cerebral insulin signaling, and this mechanism is supported by prior evidence (63–65). Thereby, it offers a novel metabolic framework for the prevention and treatment of depression. In contrast, age, D-dimer, BUN, Cr, TC, and LDL did not emerge as independent predictors of depression (all p > 0.05). This finding suggests that psychosocial factors and insulin resistance exert more direct effects on depression development in this specific population compared to these conventional clinical indicators.

Exploratory stratified analyses revealed distinct relationships among cognitive function, sleep quality, social support and the TyG index. Cognitive function and sleep quality showed no significant association with the TyG index and appeared independently related to depression. In contrast, social support was significantly associated with the TyG index in the subgroup with inadequate social support, which suggests that it may be linked to depression onset through modulation of metabolic status. These findings underscore the need for a comprehensive approach to managing depression in elderly, non-diabetic atrial fibrillation patients, combining attention to psychosocial factors and metabolic status (66). Targeted interventions, including improving cognition, optimizing sleep, and enhancing social support, may reduce depression risk and improve mental health and prognosis (67). Future research could explore potential mechanistic links among these factors to inform clinical practice.

Our findings have important clinical implications. First, identifying and managing metabolic and psychosocial risk factors may help prevent and manage depression in AF patients. Second, improving sleep quality, enhancing social support, and optimizing metabolic status may serve as effective intervention strategies. Third, clinicians should prioritize screening for and addressing emotional disorders in AF patients to improve overall health and quality of life. Clinicians can use the TyG index, CMMSE, PSQI, and SSRS as simple multidimensional assessment tools for comprehensive screening of depression risk in patients with atrial fibrillation AF. The TyG index reflects a chronic stress state associated with metabolic dysregulation. The CMMSE identifies cognitive impairment a high-risk factor for depression. While the PSQI reveals the negative impact of sleep problems on mood and the SSRS assesses the risk of isolation due to insufficient social support. In practice, it is recommended that during AF patient follow-up, TyG should be simultaneously measured and the three aforementioned scales should be administered. Then risk should be stratified based on the number of abnormal dimensions, followed by targeted interventions including optimizing metabolic and sleep management, cognitive rehabilitation training, and enhancing social support. When necessary, collaborations with a multidisciplinary team comprising cardiology, psychiatry psychology, nutrition, and social work are advised. This approach enables early identification, individualized prevention and management of depression in AF patients, which could improve the overall care quality. Although our study provides valuable insights, it has some limitations. Firstly, this is a single-center study with a cohort restricted to inpatients from one tertiary hospital in Shanghai, excluding those from community and primary care settings. Thus, the findings may have greater generalizability to inpatients in similar healthcare institutions. Additionally, collection of the patient data was incomplete. Specifically, the medication information is lacked in systematic collection. Varied medication regimens may indirectly affect patients’ psychological well-being through side effects or variations in efficacy. Moreover, socioeconomic indicators including education level, income, and healthcare payment methods could influence depression risk in AF patients by shaping health literacy and life stress levels. Consequently, future studies should address these confounding biases through more comprehensive data collection or statistical adjustment strategies. Secondly, after excluding patients with moderate to severe anxiety or a history of psychiatric disorders, the study conclusions may underestimate the depression risk or intervention needs in comorbid populations. The psychological distress caused by atrial fibrillation itself may also be a core trigger for depressive symptoms (68, 69). Our study primarily revealed clinical risks associated with the depressive symptom cluster rather than providing a precise attribution of depression etiology. Future research could integrate more precise depression-specific diagnostic instruments and enhance the external validity of findings through the implementation of multicenter designs and the incorporation of diverse patient populations with comorbid psychiatric conditions. Thirdly, the subgroup analyses conducted in this study were exploratory. For example, some subgroups exhibits small sample sizes and limited statistical power, which may lead to false-positive or false-negative results. Therefore, we do not draw definitive conclusions about subgroup differences but treat them as hypotheses to be verified in future studies. Subsequent research will further test these potential heterogeneities through pre specified stratified analyses and expanded sample sizes. This study also did not conduct formal multicollinearity diagnosis for the predictor variables included in the logistic regression model. Although the selected predictors were filtered based on clear clinical relevance and prior univariate analysis results, they were judged as relatively independent influencing factors from a clinical perspective. However, this approach cannot replace standardized multicollinearity testing. It may potentially influence the stability of model coefficients and the accuracy of interpreting results. In future studies, this step will be refined using methods including the variance inflation factor (VIF) to enhance the statistical robustness of the model. Finally, although the TyG index demonstrated statistical significance in the multivariable model, it exhibited a relatively broad confidence interval and a modest effect size. This indicated limitations regarding its predictive utility and precluded its designation as a core risk factor. As a composite derived metric of triglycerides and fasting blood glucose, the TyG index is susceptible to non-specific factors such as short-term dietary changes and fluctuations in lipid and glucose metabolism. Additionally, the baseline heterogeneity of the study population and sample size characteristics resulted in a weak independent association with the study outcome, making it difficult to establish a stable predictive effect. Furthermore, the clinical application value of the TyG index shows obvious heterogeneity across populations and study designs. Given that this study focused solely on a specific population, the external validity of the results is limited. The TyG index can serve as a potential auxiliary predictor for related outcomes in the population of this study, offering a reference for clinical risk screening.

## Conclusion

In summary, our study suggests that cognitive function, social support, sleep quality, and metabolic status are potential factors associated with an increased risk of depression in elderly non-diabetic AF patients. These observational findings may help generate the hypothesis that comprehensive management of these modifiable factors could be beneficial for improving mental health and overall prognosis in this population.

## Data Availability

The raw data supporting the conclusions of this article will be made available by the authors, without undue reservation.
